# Burnout Syndrome and Meta-Analyses: Need for Evidence-Based Research in Occupational Health. Comments on Prevalence of Burnout in Medical and Surgical Residents: A Meta-Analysis. *Int. J. Environ. Res. Public. Health*. 2019, *16*, doi:10.3390/ijerph16091479

**DOI:** 10.3390/ijerph17030741

**Published:** 2020-01-23

**Authors:** Francesco Chirico, Nicola Magnavita

**Affiliations:** 1 Post-graduate School in Occupational Health, Università Cattolica del Sacro Cuore, 00168 Rome, Italy; 2 Department of Woman and Child Health and Public Health, Università Cattolica del Sacro Cuore, Fondazione Policinico “A. Gemelli” IRCCS, 00168 Rome, Italy; nicola.magnavita@unicatt.it

**Keywords:** burnout syndrome, evidence-based research, meta-analysis, observational studies, systematic review

## Abstract

In their meta-analysis of observational studies, Low et al. showed a high prevalence of burnout syndrome (BOS) among medical and surgical residents across the globe with an aggregate prevalence of burnout as 51.0% (CI: 45.0–57%). However, the sample size in many of the included studies was quite low (only 26 out of 47 included studies had a sample size of more than 100 participants), and almost all of the 47 studies reported a rate of respondents of less than 80% (43 out of 47, 91.4%). Furthermore, in many of them, the rate of respondents was unknown (5 out of 47) or less than 50% of eligible persons (23 out of 47 studies). As BOS is a self-reported syndrome, healthcare professionals who decided to participate in those studies were many of those affected by BOS, making the percentage of respondents potentially overstated due to the nonresponse bias. Policy decision-making in public health relies on evidence-based research; therefore, quality evaluation of studies in meta-analysis is essential to draw useful data for policymakers.

Policy decision-making in public health relies on evidence-based research. Burnout syndrome (BOS) is a worrying phenomenon among clinicians and residents, not only in the United States, but across the entire globe [1]. However, systematic reviews and meta-analyses on prevalence of burnout syndrome in physicians often lead to contradictory results given the difficulty of defining and measuring with precision this puzzling syndrome. Despite this, burnout syndrome, whose diagnostic criteria remain uncertain, is increasingly recognized and diagnosed by occupational stakeholders to such an extent that in many countries, this syndrome is widely being considered as an occupational disease [2,3,4].

Therefore, the meta-analysis [5] of observational cohort and cross-sectional studies carried out by Low et al., reporting the prevalence of burnout syndrome measured with the Maslach Burnout Inventory among medical residents, is absolutely valuable as an effort to fill a deep gap of knowledge in an occupational sector where costs and consequences of burnout syndrome may be very relevant, as the quality of cure delivered by healthcare professionals may be compromised [6,7].

In every systematic review and meta-analysis, however, the quality evaluation of included studies is essential to drawing useful data to be translated by policymakers into public health decisions. We have noted in this meta-analysis that Low et al. adopted the National Institute of Health’s Quality Assessment Tool for Observational Cohort and Cross-Sectional Studies (NIH-QAT) [8], which is a tool to assess various aspects of a study by assigning to the study an overall quality rating of “Good”, “Fair”, or “Poor”. However, one of this scale’s criteria considers the rate of study participants to be at least 50% of eligible persons, whereas other well-recognized and most-used tools, such as the Newcastle–Ottawa Scale [9] for cohort and case-control studies, put this percentage at 80%. We explored the overall rate quality of the studies included and shown in Appendix 1 by authors. In Table 2, the prevalence of burnout of studies included by authors is high, making an aggregate prevalence of burnout as 51.0% (CI: 45.0–57%), but the sample size in many studies is quite low (only 26 out of 47 included studies had a sample size of more than 100 participants), and almost all of the 47 studies reported a rate of respondents of less than 80% (43 out of 47, 91.4%). Furthermore, in many of them, the rate of respondents was unknown (5 out of 47) or less than 50% of eligible persons (23 out of 47 studies).

As BOS is a self-reported syndrome, participants who responded were probably also those affected by BOS, potentially making the percentage of respondents higher due to the nonresponse bias; that is, the error resulting from distinct differences between the people who responded to a survey versus the people who did not respond [10]. The prevalence of burnout, therefore, could even be overstated. Quality evaluation of studies in meta-analysis is essential to draw useful data for policymakers and it highlights a very significant limitation of the review to be taken into account, while making targeted interventions of public health policies that necessitate cost-effectiveness.
